# The TRiCky Business of Protein Folding in Health and Disease

**DOI:** 10.3389/fcell.2022.906530

**Published:** 2022-05-05

**Authors:** Heba Ghozlan, Amanda Cox, Daniel Nierenberg, Stephen King, Annette R. Khaled

**Affiliations:** ^1^ Division of Cancer Research, Burnett School of Biomedical Sciences, College of Medicine, University of Central Florida, Orlando, FL, United States; ^2^ Department of Physiology and Biochemistry, Jordan University of Science and Technology, Irbid, Jordan; ^3^ Division of Neuroscience, Burnett School of Biomedical Sciences, College of Medicine, University of Central Florida, Orlando, FL, United States

**Keywords:** cytoskeleton, cell cycle, cancer, neurological disorder, chaperonin, proteostasis

## Abstract

Maintenance of the cellular proteome or proteostasis is an essential process that when deregulated leads to diseases like neurological disorders and cancer. Central to proteostasis are the molecular chaperones that fold proteins into functional 3-dimensional (3D) shapes and prevent protein aggregation. Chaperonins, a family of chaperones found in all lineages of organisms, are efficient machines that fold proteins within central cavities. The eukaryotic Chaperonin Containing TCP1 (CCT), also known as Tailless complex polypeptide 1 (TCP-1) Ring Complex (TRiC), is a multi-subunit molecular complex that folds the obligate substrates, actin, and tubulin. But more than folding cytoskeletal proteins, CCT differs from most chaperones in its ability to fold proteins larger than its central folding chamber and in a sequential manner that enables it to tackle proteins with complex topologies or very large proteins and complexes. Unique features of CCT include an asymmetry of charges and ATP affinities across the eight subunits that form the hetero-oligomeric complex. Variable substrate binding capacities endow CCT with a plasticity that developed as the chaperonin evolved with eukaryotes and acquired functional capacity in the densely packed intracellular environment. Given the decades of discovery on the structure and function of CCT, much remains unknown such as the scope of its interactome. New findings on the role of CCT in disease, and potential for diagnostic and therapeutic uses, heighten the need to better understand the function of this essential molecular chaperone. Clues as to how CCT causes cancer or neurological disorders lie in the early studies of the chaperonin that form a foundational knowledgebase. In this review, we span the decades of CCT discoveries to provide critical context to the continued research on the diverse capacities in health and disease of this essential protein-folding complex.

## Introduction

Keeping proteins in the correct shape and properly folded depends on balanced protein homeostasis that is essential for the growth and survival of cells. Failure of the quality control mechanisms that maintain the cellular proteome and regulate the synthesis, folding, trafficking, and degradation of proteins is a hallmark of disease. Neurodegenerative disorders may be a consequence of proteostasis deficiency, while cancer could result from enhanced proteostasis capacity (Powers et al., 2009). Key structural studies of molecular chaperones in the 1990s (Marco et al., 1993) revealed these as essential components of the network governing protein homeostasis. Though *in vitro* studies revealed much on the basic principles of protein folding, how proteins fold in the crowded *in vivo* cellular environment remains to be fully understood. Most small proteins spontaneously fold (Anfinsen, 1973), while large proteins that represent the majority of a cellular proteome depend on molecular chaperones to reach their native state (Bartlett and Radford, 2009).

Chaperome refers to the collective cellular folding machinery that includes chaperone, co-chaperone, and chaperonin (Wang et al., 2006). Often chaperones are activated by stress; for example, heat shock proteins (HSPs) are chaperones first described in response to heat shock (Lindquist and Craig, 1988). Major chaperone classes are categorized by their molecular weight, like HSP40, HSP70, or HSP90, and have functions, such as *de novo* protein folding, that support proteostasis (Hartl et al., 2011). Chaperonins are multi-component complexes [∼800–900 kilodalton (kDa)] that couple protein folding with adenosine 5′-triphosphate (ATP) hydrolysis or partner with co-factors like small HSPs and also prevent protein aggregation. Two major groups of chaperonins, group I and group II, are found in all lineages of organisms (bacteria, archaea, eukaryotes). The best studied group I chaperonin is GroEL-Gro-ES found in *Escherichia coli* (*E. coli*). The group II chaperonins include the archetypal *Thermoplasma acidophilim* α/β-thermosome and *Methanococcus maripaludis* chaperonin (Mm-Cpn) and the eukaryotic chaperonin, CCT, also known as TRiC. CCT, as is referred to herein, is a hetero-oligomeric chaperonin with a cylindrical structure composed of two stacked rings of eight subunits. While initial studies suggested that CCT folds 9–15% of the cellular proteome (Thulasiraman et al., 1999), other studies report that 7% or as little as 1% of the proteins in cells are CCT substrates (Yam et al., 2008; Willison, 2018). Indicative of its importance, deregulation of CCT is observed in cancer (Boudiaf-Benmammar et al., 2013; Roh et al., 2015; Vallin and Grantham, 2019), infectious diseases (Inoue et al., 2011; Bugnon Valdano et al., 2021) and neurodegenerative disorders (Pavel et al., 2016; Sot et al., 2017; Chen, 2019), suggesting a central role in the pathogenesis of these conditions. The early decades of CCT discoveries were focused on understanding CCT function, expression, and evolution. Obligate substrates, like actin and tubulin, were reported to interact with CCT along with a few non-cytoskeletal proteins. In the last decade, however, advances in proteomics and investigations into the role of CCT in disease prompted the discovery of new substrates and new activities. In this review, we will bridge discoveries from different eras to better understand the role of CCT in health and disease, addressing how CCT is formed and regulated, and in turn, modulates cellular processes through its distinctive structure and function.

### The Evolution of Chaperonin Containing TCP1 in the Crowded Cellular Environment

#### The Structure of Chaperonin Containing TCP1 and Subunit Arrangement

**FIGURE 1:**
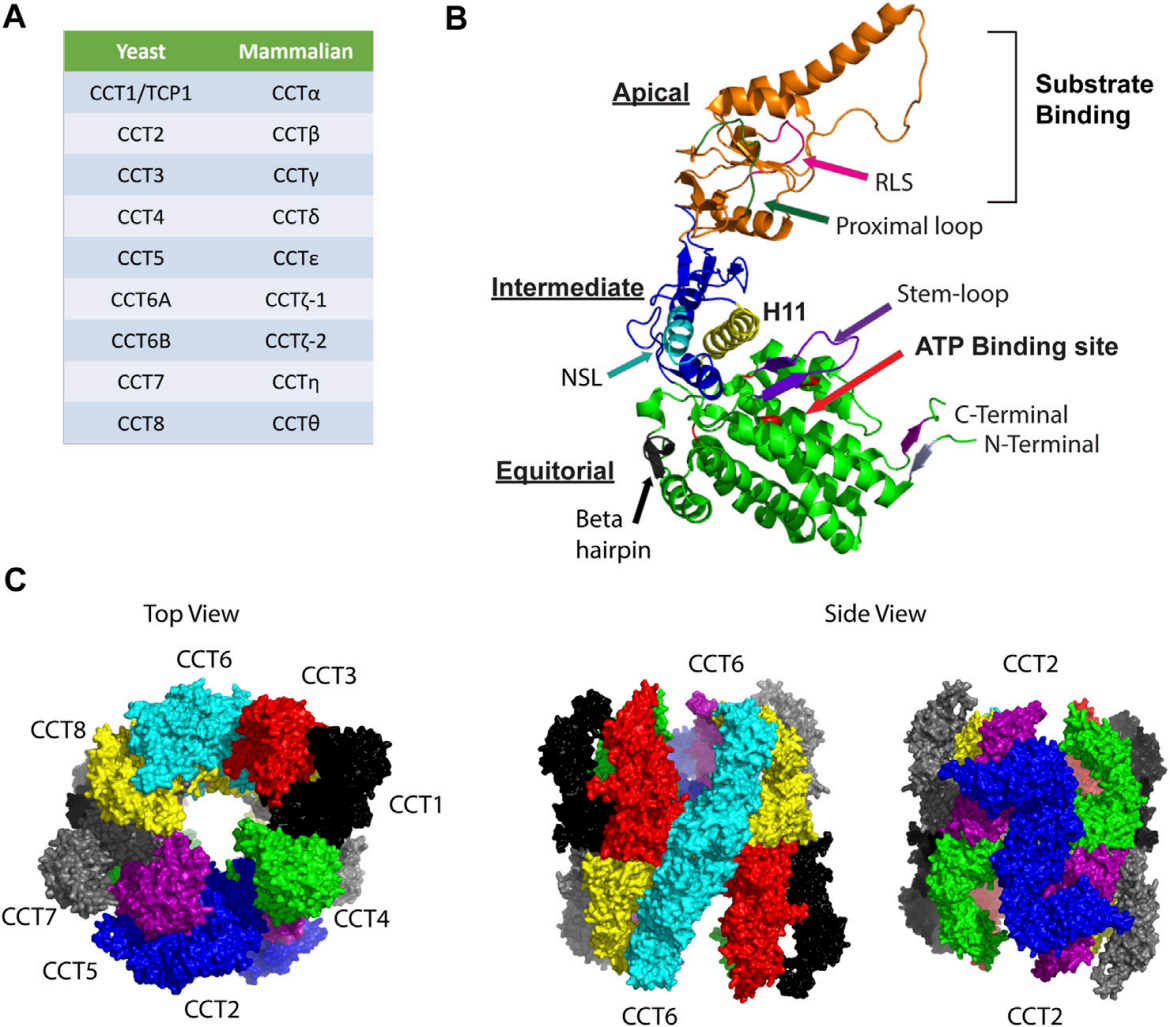
Organization of the CCT complex. **(A)** Nomenclature for CCT subunits based on yeast and mammalian studies. **(B)** Model for structure of a CCT subunit with the major structural elements indicated in the apical, intermediate, and equatorial domains. NSL, nucleotide sensing loop; RSL: release loop for substrate. The model was made based on PDB:3KTT [atomic model of bovine CCT2 (Cong et al., 2010)]. **(C)** Ring structure organization of CCT subunits is shown based on PDB 5GW4 [Structure of Yeast NPP-TRiC (Zang et al., 2016)]. Top view shows the open ring conformation with subunits indicated as follows: CCT1 (black), CCT2 (blue), CCT3 (red), CCT4 (green), CCT5 (purple), CCT6 (cyan), CCT7 (grey), and CCT8 (yellow). Side views highlight the homotypic interactions between CCT2-CCT2 (blue) and CCT6-CCT6 (cyan).

CCT is a multi-oligomeric, double torus complex composed of eight subunits, termed CCT1-8 (yeast) or CCTα-θ (mammals) (Rommelaere et al., 1993; Kubota et al., 1994) (Figure 1A). Evolution of the eukaryotic chaperonin is the result of gene duplication and gene loss events that remain ongoing processes. The eight paralogous CCT subunits share high amino acid similarity (27–39% sequence identity) (Archibald et al., 2001), whereas CCT orthologues are conserved across species. Phylogenetic analysis suggested that different CCT subunit genes resulted from duplication events that happened early in the evolution of eukaryotes, then independently diverged to have a unique function that is maintained in most eukaryotes. CCTδ (CCT4), CCTε (CCT5), CCTα (TCP-1/CCT1), CCTβ (CCT2), and CCTη (CCT7) are the most recent results of gene duplication events (Kubota et al., 1994; Archibald et al., 2000; Archibald et al., 2001; Fares and Wolfe, 2003). CCT polypeptides vary in length from 531 to 556 residues, and in molecular weights from 52 to 65 kDa (Kubota et al., 1994). The ATP-binding domain is the most conserved across CCT subunits, while the substrate-binding domain varies across subunits (Kim et al., 1994; Kubota et al., 1994; Archibald et al., 2000).

Each CCT subunit assembles in a precise ring arrangement to form a cylindrical barrel with an enclosed chamber that supports a hydrophobic environment where substrate folding takes place. Each CCT subunit has three different domains: an apical domain that encloses the chamber and forms the substrate-binding site, an equatorial domain that forms the wall of the folding chamber, is involved in ring-ring interactions, and contains the ATP binding site, and an intermediate domain that connects the apical and equatorial domains (Figure 1B). Reports using a combination of methods (chemical cross-linking, mass spectrometry, and combinatorial modeling) revealed that CCT subunits are arranged in the ring in the following order: CCT4-2-5-7-8-6-3-1 (Leitner et al., 2012). The double ring complex displays a two-fold symmetry in which subunits CCT2 and CCT6 form a homotypic interface while the other subunits contact points on the heterotypic form (Figure 1C) (Leitner et al., 2012). The N-and C-termini of CCT subunits are buried inside the barrel to form a septum between the two chambers (Dekker et al., 2011b; Kalisman et al., 2013), with the exception of the N-terminus of CCT4 that protrudes towards the outside of the complex. This structural arrangement is evolutionally conserved as CCT4 is the only subunit with a conserved proline residue in the outward pointing N-terminus. The inner walls of the CCT folding chamber are hydrophilic, with a bipolar surface-charge distribution that is conserved as well, with the CCT5-2-4 side being positively charged while the CCT3-6-8 side is negatively charged (Leitner et al., 2012). The asymmetry of charge is among the features of CCT that may have evolved to provide specific solutions to the folding of proteins with complex topologies and assembly of large multi-protein structures.

#### The Assembly, Disassembly, and Degradation of Chaperonin Containing TCP1

The specific order of subunit assembly could be critical in achieving the desired function of a multi-component complex. An example is the Biedel Bradet Syndrome (BBS) subunits forming the BBSsome that is involved in ciliary membrane biogenesis. Assembly of BBS some is dictated by specific protein-protein interactions involving CCT subunits, and regulation could be at the level of the BBS-chaperonin complex formation (Zhang et al., 2012). Whether CCT assembles in a similar manner is unknown. Early studies involving the purification of CCT from yeast (Dekker et al., 2011a), bovine tissue (Ferreyra and Frydman, 2000), or mouse testes (Knee et al., 2013) advanced understanding of the structure and function of the chaperonin but provided few insights on assembly of the complex. The presence of CCT micro-complexes, ranging from 120 to 250 kDa, were detected in cells along with the intact hetero-oligomer, but were minor species and their importance was unclear (Liou and Willison, 1997). Subsequently, single CCT subunits were expressed in *E. coli* and sedimentation of CCT subunits by a sucrose gradient revealed that two of the CCT subunit proteins, CCT4 and CCT5, formed functional homo-oligomeric rings, while other subunits, CCT2, CCT3, CCT7, and CCT8, were observed as slowly sedimenting species suggestive of monomers, and CCT1 was bound to the ribosome (Sergeeva et al., 2013). Data from structural studies showing that CCT complexes usually contained all eight subunits, even when reconstituted from eight plasmids (Machida et al., 2012), indicated that CCT4 and CCT5 homo-oligomers may not have activities similar to hetero-oligomer, but could have intermediate functions, for example in the assembly process.

Expression data from early studies of CCT suggested that there was equal stoichiometry of CCT at the mRNA and protein level (Rommelaere et al., 1993; Kubota et al., 1999b). However, a body of evidence later suggested that some CCT subunits could be overexpressed under certain conditions, such as cancer cell proliferation (Boudiaf-Benmammar et al., 2013) or cell cycling (Yokota et al., 2001b). Hence, it is possible that transient populations of CCT-complexes composed of select subunits could exist. CCT4 and CCT5 subunits formed functional homo-oligomers in *E. coli* (Sergeeva et al., 2013), and CCT5 homo-oligomers had a similar structure as the hetero-oligomeric CCT complex (Pereira et al., 2017). The existence of monomeric CCT subunits in cells was reported in multiple studies, and these subunits may interact with other proteins. Hence, monomeric CCT subunits could incorporate into CCT4 or CCT5 homo-oligomers as an initiating step of complex assembly. This was tested in an *E. coli* model system in which CCT subunits were individually expressed with either CCT4 or CCT5 homo-oligomers to determine which CCT subunits were more likely to hetero-oligomerize with CCT4 or CCT5-based complexes (Sergeeva et al., 2019). With the exemption of CCT6, all CCT subunits were present in complexes when co-expressed with the CCT5 homo-oligomer, while the CCT4 homo-oligomer was the least effective in complex interactions, binding only CCT5 and CCT8. Based on these subunit-subunit interactions, sedimentation patterns, and previous structural data, a model for CCT complex assembly was proposed starting from the CCT5 homo-oligomer as a template or intermediate form. CCT subunits assemble onto the CCT5 homo-oligomeric ring in the order of CCT2 first, followed by CCT4, CCT1, CCT3, CCT7, CCT8, and lastly CCT6 (Sergeeva et al., 2019). This idea is supported by mass spectrometry analysis of human CCT purified from insect cells, in which CCT5 was the most stable subunit observed under chemical destabilization conditions, hence, the most likely to self-assemble or co-assemble with other subunits. Importantly, CCT5 formed dimers with all subunits except for CCT8 (Collier et al., 2021). However, since CCT subunits are independently transcribed in the crowded cellular environment, bringing together all the subunits for assembly could be challenging. Post-translational modifications (PTMs) that sequester CCT subunits in subcellular compartments would be a solution. S-palmitoylation is a PTM in which palmitic acid is attached to cysteines of proteins through a thioester bond, resulting in increased local hydrophobicity that drives associations with membranes and well as protein-protein interactions. Interestingly, most of the CCT subunits were found to be S-palmitoylated, with S-palmitoylation of CCT1, CCT2, CCT3, CCT4, and CCT5 experimentally proven (Blanc et al., 2015). Since S-palmitoylation is dynamic and reversible, it is intriguing to speculate that this PTM may help localize CCT subunits to membranes, like the endoplasmic reticulum, and facilitate the ordered assembly of CCT subunits onto a CCT5 template to form the hetero-oligomeric complex (Sergeeva et al., 2019).

Disassembly and degradation of the CCT complex are processes less well understood than assembly. Early studies performing *in vitro* translation reactions for the eight CCT subunits suggested that ring disassembly may not be a global process but rather occurs in one ring at a time during each protein-folding cycle to yield micro-complexes and monomers (Liou et al., 1998). Kinetics of the process further suggested that in the absence of a pre-existing template, assembly of the CCT complex would be too slow. Hence, a semiconservative model based on the disassembly of a single ring is likely to be thermodynamically favorable (Liou et al., 1998). Dissociation of CCT complexes into smaller micro-complexes and monomers was also observed under conditions of physiological levels of potassium (K+) and ATP (Roobol et al., 1999a). Implications are that CCT turnover may actively occur during the ATP-driven folding cycle. Performing immunoprecipitation of CCT using the neuroblastoma/rat dorsal ganglion hybrid cell line (ND7/23) revealed that ATP/K+ caused precipitation of monomeric CCT subunits, while CCT precipitated as the intact hetero-oligomeric complex in the absence of ATP. Interestingly, the addition of the non-hydrolyzable ATP analog, AMP-PNP, was not effective in precipitating free CCT subunits; therefore, ATP hydrolysis was important for the observed effect of subunit dissociation. The disassembly of subunits from the CCT complex occurred most readily with CCT2 and CCT8, followed by CCT1, CCT4, CCT6, and CCT5, with CCT3 being last. Homology analysis revealed that the length of the loop connecting the equatorial and intermediate domain for each CCT subunit correlated with the CCT precipitation profile in the presence of ATP. The longest loops were found in CCT3 and the shortest in CCT2 (Roobol et al., 1999a), which could control how CCT subunits exit the chaperonin complex. N-terminal modifications may also affect the formation of the CCT complex since the N-termini of subunit apical domains mediate inter-ring cooperativity. Truncation of N-termini through methionine loss or acetylation could lead to degradation of CCT subunits (Collier et al., 2021). Evident from these experiments is that assembly/disassembly of the CCT complex may be linked to the monomer/oligomer pool in a dynamic process that ultimately controls protein folding activity and critical cell functions.

Pulse-chase experiments with mammary carcinoma FM3A cells revealed that turnover rates of individual CCT subunits varied significantly, with CCT4 having the shortest half-life (∼4 h) and CCT2 the longest half-life (∼8 h) (Yokota et al., 2001b). A systematic analysis of protein half-lives in MCF-7 breast cancer cells found, in contrast, that CCT4 had the longest half-life (∼10.6 h), followed by CCT8, CCT7 and CCT2, with half-lives between 7–8 h, and CCT5 and CCT3 with the shortest half-lives of 4.6 and 2.5 h, respectively (Tong et al., 2020). Such results suggest that, in addition to ATP hydrolysis controlling disassembly of the complex, variable turnover rates of CCT subunits could be cell type specific, limiting subunit availability and formation of the active chaperonin. Degradation of the CCT complex through the ubiquitin-proteasome system was shown using the proteasome inhibitor, lactacystin, as well as with temperature-sensitive mutations in the ubiquitin-activating enzyme E1 (Yokota et al., 2000a). CCT5 also associated with the 26S proteasome (Tokumoto et al., 2000). In MCF-7 cells treated with the proteasome inhibitor, bortezomib, the half-lives of CCT subunits doubled, with some, like CCT4, reaching a half-live of almost 20 h (Tong et al., 2020). Another quality control mechanism that could be triggered by CCT with unfolded actin is autophagy, a system for degradation and re-utilization of intracellular contents. Using a CCT1/TCP-1-RFP-GFP fusion protein as a readout, where GFP is pH sensitive, the degradation of CCT in lysosomes during autophagy was observed when actin dynamics were perturbed. While the mechanisms regulating the autophagic degradation of CCT remain to be determined, a link between CCT, actin homeostasis, and autophagy is possible and could be triggered by conditions that occur, for example, during cell cycle arrest (Date et al., 2022).

#### The Chaperonin Containing TCP1 ATPase Cycle

In most chaperonins, ATP binding and hydrolysis are linked to substrate folding in an ordered process; however, in CCT the mechanism is more complex. Unlike Gro-EL in which the co-factor, Gro-ES, functions as a detachable lid, group II chaperonins have a built-in lid formed by protrusions in the apical domains of each subunit. Subunit equatorial domains bind ATP through a conserved phosphate-binding or P-loop motif, while ATP hydrolysis is triggered by a catalytic aspartic acid residue located in the subunit intermediate domains. CCT can be in a lid open state, when substrate binding sites are accessible, or a lid closed state where the apical segments of the lid come together to form a beta-stranded iris (Llorca et al., 1999b; Gutsche et al., 2000; Cong et al., 2012; Zang et al., 2016). The transition between the open and closed states of CCT is referred to as the ATPase cycle. Insights on the chaperonin ATPase cycle first came from studies of Mm-Cpn that were later confirmed for CCT. In the nucleotide-free state, CCT is open, with subunit apical domains in different conformations. ATP binding alone is not enough to induce the closed state but does promote changes in the apical domains that generate a more compact open conformation called a tetramer of dimers that creates a pseudo four-fold symmetry first described in bovine CCT (Cong et al., 2012). Upon hydrolysis of ATP, triggered by the catalytic aspartic acid residue, a conformational change in the apical domains leads to closing of the lid and movement of the substrate into the central chamber where folding occurs. Subsequent release of phosphate and ADP end the cycle, re-opening the chamber to release substrate (Gestaut et al., 2019a).

High-resolution cryogenic electron microscopy (cryo-EM) revealed the structure of CCT in different states of nucleotide occupancy during the ATPase cycle. CCT structures reported in the decade between 1999–2010, using nucleotide analogs, showed possible models for arrangements of the eight subunits in a ring, with or without substrates (Llorca et al., 1999b; Llorca et al., 2001; Booth et al., 2008; Cong et al., 2010). But it was single particle cryo-EM studies of bovine CCT in the apo (nucleotide-free), ATP-bound, ADP-bound, and ATP-hydrolysis transition states that revealed mechanisms distinct from other group I or II chaperonins (Cong et al., 2012). The apical domains of CCT subunits in the apo, ATP- and ADP-bound states extended towards the central folding chamber at different angles but tilted upwards and open in the ATP-bound state, re-affirming that ATP binding did not close the lid. Moreover, in the open states, contacts between the apical domains created the open and more compact configuration termed the tetramer of dimers. The structure of CCT in these three states suggested that the ADP-bound state could be the substrate acceptor state. Some structures from the ATP transition states were of a hybrid or asymmetric conformation in which the *cis* ring was closed and the *trans* ring was open, suggestive of negative cooperativity and that CCT functioned like a two-stroke motor (Reissmann et al., 2007; Cong et al., 2012). However, recent cryo-EM structures of yeast CCT noted the synchronous hydrolysis of ATP that was indicative of positive inter-ring cooperativity in which both rings were closed (Jin et al., 2019), suggesting that nucleotide binding in both rings followed the same order.

**FIGURE 2:**
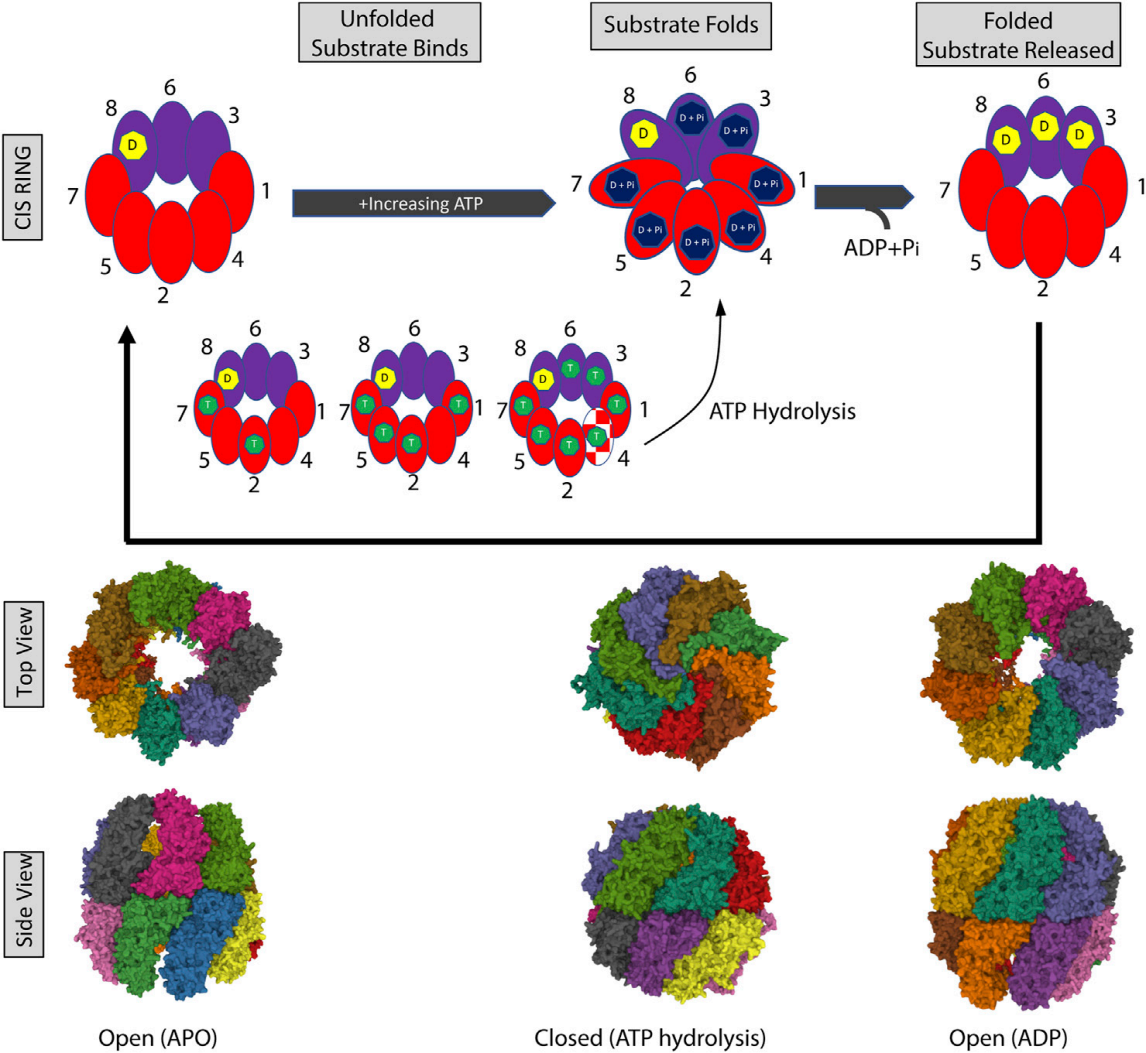
The CCT ATPase cycle. (A) Schematic shows the sequential binding of ATP starting with subunits on the CCT2 side of the complex (red) followed by the CCT6 side of the complex (purple). CCT7 and CCT2 first react to and bind nucleotide, followed by the rest of the subunits with increasing ATP. CCT4 (red/white pattern) is the last to bind to ATP and this binding triggers ATP hydrolysis and closing of the ring. CCT3 and CCT6 may load with ATP later in the cycle (delayed release of ADP), while the ADP bound to CCT8 may be exchanged for ATP only under high nucleotide concentrations and/or not involved in ATP consumption. The processing of an unfolded and folded substrate is shown in the gray boxes. ADP (D) is indicated by the yellow heptagon, ATP by the green heptagon, and hydrolyzed ATP: ADP + phosphate (D + P_i_) in the dark blue heptagon. The nucleotide-free open ring conformation is based on PDB 4A0O [nucleotide-free (apo) state (Cong et al., 2012)]. The closed ring conformation is based on PDB 6KS6 [Yeast CCT at 0.2 mM ADP-AlFx (Jin et al., 2019)]. The open ring conformation with ADP is based on PDB 4A13 [model refined against symmetry-free cryo-EM map of TRiC-ADP (Cong et al., 2012)].

Unlike the archaeal chaperonins, the ATPase cycle in CCT is highly asymmetric. Genetic studies and biochemical studies showed that CCT subunits have different ATP affinities—with four to five subunits strongly binding ATP (Reissmann et al., 2012; Zang et al., 2016). Hence a gradient of ATP affinities exists, with CCT5 and CCT4 having the highest affinities, followed by CCT2 and CCT1. The remaining subunits, especially CCT3, CCT6, and CCT8, have a low affinity for ATP. Using yeast as a model system to evaluate mutations introduced into the CCT subunits, mutations in the P-loop or the catalytic aspartic acid of the high ATP-affinity subunits (CCT7, 5, 4, 2) caused loss of growth. In contrast, the same mutations introduced into the low ATP affinity subunits (CCT8, 6, 3) did not cause loss of growth (Amit et al., 2010; Reissmann et al., 2012), with further work showing that these subunits may have very slow ADP off-rates (Zang et al., 2016). This suggests the existence of a staggered ATP-binding mechanism, which was shown by cryo-EM using single subunit eGFP tags. The subunits on the CCT2 side of the complex might initiate ATP binding, while the subunits on the CCT6 side might bind ATP later due to the delayed release of ADP (Zang et al., 2016) (Figure 2). Importantly, these studies indicate that the ATPase cycle of CCT is responsive to the concentration of ATP. Kinetics-based calculations inferred that the ATPase reaction in CCT reaches a plateau at 0.2 mM ATP with most subunits binding nucleotides (Shimon et al., 2008). It follows that at lower concentrations of ATP not all subunits will bind nucleotides. Resolution of the structure of yeast CCT at low concentrations of ATP (near cell starvation levels), revealed that CCT7, followed by CCT2, first reacted to nucleotide binding and may initiate ring closure, while the other subunits bound ATP as levels increased. CCT4 was the last subunit to bind ATP and could be an ATP sensor, triggering ATP hydrolysis only when the ATP concentration reaches cell sustainable levels (Figure 2). This is supported by previous studies showing the presence of a dynamic β-hairpin motif in the nucleotide-binding site of CCT4 (Zang et al., 2016) and the fact that loss of the ATP hydrolysis activity in CCT4 was lethal in yeast (Amit et al., 2010). CCT8 was also unique in that it remained bound to ADP longer than other subunits and may have a role independent of ATP hydrolysis, through its N-terminus, in CCT allosteric cooperativity (Jin et al., 2019) and complex assembly (Noormohammadi et al., 2016). Further, the dynamic CCT2 intra-ring interactions between the apical domains of CCT5 and CCT4 and the strong CCT2-CCT2 inter-ring interaction upon ATP binding also suggest an important role for CCT2 in CCT allosteric cooperativity (Zang et al., 2016).

The folding of substrate is dependent on the ATPase cycle. Structural studies revealed that the interface of substrate binding to the apical domain of chaperonin subunits involved a groove between Helix 11 (H11) and a proximal loop (PL) that contains a mix of charged, polar, and hydrophobic residues (Joachimiak et al., 2014) (Figure 1B). Shown in Mm-Cpn, ATP hydrolysis results in a conformational change that brings H11-PL into proximity of a loop in an adjacent subunit, with subsequent packing displacing the substrate and pushing it into the chamber for folding (Douglas et al., 2011). In studies with CCT, denatured actin (with some secondary and tertiary structure acquired) bound to the high ATP-affinity CCT2 side of the chaperonin, which kept the two lobes of actin extended and prevented misfolding. To achieve the native state of actin, ATP binding and subsequent hydrolysis triggered the segmental release of bound actin into the folding chamber (Balchin et al., 2018), bringing the actin lobes together. Hence, by controlling the order of folding through the ATPase cycle, CCT lowers the folding energy barrier for actin and similar proteins with complex topologies through the formation of high contact order interactions not possible with the simpler chaperonins. This sequential ATP-induced conformational change may also govern the cycles of protein folding and the subsequent release of multidomain proteins during the folding process (Rivenzon-Segal et al., 2005; Reissmann et al., 2012).

#### Substrate Folding by Chaperonin Containing TCP1

Substrates folded by CCT are structurally and functionally diverse, driving the evolution of a complex chaperonin with diversification of subunits that can modulate substrate binding specificities. The apical domains of CCT subunits are the principal sites for substrate recognition, but exact structures or sequence rules that regulate substrate binding may depend on the conditions of protein synthesis and cooperation of co-chaperones. A comparison of substrates folded by CCT under *in vitro* compared to *in vivo* conditions revealed little overlap, other than actin and tubulin, between the two groups of proteins, suggesting that substrate binding to CCT depended on factors beyond specific amino acid sequences (Yam et al., 2008). As an example, patterns of polar and hydrophobic residues in the groove between H11 and PL in the apical domain of CCT subunits may govern substrate binding affinities through combinatorial interactions (Joachimiak et al., 2014). While the field is undecided on the size of the intracellular protein pool folded by CCT and the scope of the CCT interactome, early studies recognized the cytoskeletal proteins, actin and tubulin, as obligate substrates of the chaperonin complex (Sternlicht et al., 1993; Llorca et al., 1999a; Roobol et al., 1999b; Hynes and Willison, 2000; Llorca et al., 2000). Non-skeletal substrates include proteins from the 7-bladed WD40 propeller repeat family (Valpuesta et al., 2002; Kubota et al., 2006). WD40 proteins form the core of substrates conserved from yeast to human that are folded by CCT (Willison, 2018). Structural data suggested that the size of the folding chamber in CCT was equivalent to GroEL-GroES (Cong et al., 2010; Cong et al., 2012) and would fold proteins up to ∼70 kDa. However, CCT is known to fold proteins that are greater than 70 kDa. Studies using fusion proteins (e.g., actin-GFP) with *in vitro* translation systems found that CCT fully encapsulated substrates smaller than its folding chamber, like actin, and partially encapsulated large multidomain proteins like the 109-kDa spliceosomal U5 subunit, hSnu114; a beta-strand rich protein. CCT partially enclosed the C-terminal domain of this protein, while the N-terminal domain was CCT-independent (Rüßmann et al., 2012). CCT thus has the capacity to fold large multi-domain proteins in a sequential manner that is domain-specific, and this may explain its role in preventing aggregation of proteins with polyglutamine (polyQ) tracts (Nollen et al., 2004). The capacity of CCT to fold proteins with complex topologies is further supported by cooperative folding with co-chaperones, like HSP70 (Cuellar et al., 2008; Stein et al., 2019), phosducin-like proteins (PhLP) (Martin-Benito et al., 2004; Stirling et al., 2006), and prefoldin/GimC (PFD) (Siegers et al., 1999).

Actin (*α*, *β*, and *γ* isoforms) is a highly conserved protein among eukaryotes that exists as free G-actin monomers and F-actin polymers, depending on ionic concentrations and ATP. The actin monomer consists of two domains, originally called “large” and “small” domains. During folding, nascent actin binds two to three CCT subunits using a 1.4 geometrical configuration (numbering based on the position of the subunits in the ring) in an open conformation, starting with subunits opposite to CCT4 in the ring that are nucleotide-free or ADP-bound (Llorca et al., 1999b; Llorca et al., 2000). ATP binding then drives the power stroke movement that leads to actin folding. The folding kinetics of actin are biphasic, with the initial folding phase being the most efficient. The topology of the CCT-bound actin facilitates rapid folding during the first phase in concert with ATP binding and hydrolysis that triggers an asymmetric conformational change in the ring structure (Balchin et al., 2018). During the second phase, the assistance of the co-chaperone, PFD, may enhance the rate and yield of actin folding. PFD is a ∼100 kDa chaperone consisting of two beta-subunits that assist actin and tubulin folding by CCT. Through a conserved electrostatic interaction, PFD interacts with CCT in the open state, aligning the substrate binding regions of the two chaperones and optimizing the substrate environment to facilitate actin folding and prevent aggregation (Gestaut et al., 2019b). In contrast, another co-chaperone, PhLP3, may inhibit CCT folding activity by forming a ternary complex with chaperonin along with actin or tubulin. The interaction of PhLP3 with CCT in the presence of substrate inhibited ATPase activity and affected the folding of nascent actin (and tubulin) *in vitro* (Stirling et al., 2006). Together, the antagonistic actions of PFD and PhLP3 may help modulate the activity of CCT to regulate the formation of the actin cytoskeleton and the biogenesis of tubulin in order to meet cellular demands.

The transcription of actin is regulated through the myocardin-related co-transcription factor-A (MRTF-A)/serum response factor (SRF) pathway. When cellular signaling receptors stimulate actin polymerization, G-actin levels decrease and disrupt the MRTF-A: actin interaction, allowing MRTF-A to move to the nucleus where it activates SRF to transcribe more actin. However, the impediment in producing actin is not at the level of transcription, but rather at the protein folding step through CCT. To regulate this, the monomeric form of CCT5 was reported to alter the nuclear accumulation of MRTF-A during serum stimulation, delaying the transcription of actin. Monomeric CCT5 accumulated when levels of the CCT hetero-oligomeric complex were low and decreased when levels of the oligomer were high (Elliott et al., 2015). Linking actin transcription and folding through CCT5 ensures that actin is synthesized only when enough CCT hetero-oligomeric complex is present to efficiently fold the cytoskeletal protein, else the presence of CCT5 monomers delays actin transcription. While free CCT monomers had been previously described (Liou and Willison, 1997; Brackley and Grantham, 2010), this report connects CCT5 monomer activity with the protein-folding function of the hetero-oligomeric chaperonin in the context of actin folding (Elliott et al., 2015).

Early studies, based on 3D reconstruction using cryo-EM, revealed that tubulin (*α*, *β*, and *γ* families) interacts with CCT differently from actin, with a mixture of shared and unique subunits used for binding (Llorca et al., 1999b). Tubulin binds up to five CCT subunits using a 1.5 geometrical configuration in a semi-closed conformation that spans the protein-folding chamber (Llorca et al., 2000). Tubulin binds to a cluster of high ATP-affinity and low ATP-affinity CCT subunits, engaging with electrostatic and polar side chains on residues (Pappenberger et al., 2002). Molecular dynamics simulations of single subunit interactions with substrate revealed that tubulin binds to CCT3 through an interface that spans the HL (helical region) and HP (helical protrusion) sites in the apical domain. This CCT3-tubulin interaction involved hydrophobic and electrostatic interactions on the HL site and a salt bridge network between charged amino acids on the HP. Generalizing this finding suggests that substrates interacting with CCT *via* hydrophobic interactions may use the HL binding sites, while substrates, like tubulin, that interact with CCT *via* electrostatic interactions, contact both HL and HP sites, with the salt bridge network being critical (Jayasinghe et al., 2010). Importantly, having obligate substrates bind to multiple CCT subunits based on geometric configurations may be advantageous in applying the mechanical force needed to fold substrates independently of having common features. Actin and tubulin are different proteins, yet both can be folded by CCT based on distinct geometric conformations (Vallin and Grantham, 2019). CCT may also fine-tune its protein-folding activity by interacting with partners. The binding of β-tubulin to CCT was modulated through interaction with Programmed cell death protein 5 (PDCD5). Cryo-EM studies showed that PDCD5 attached to the apical domain of CCT2, which sterically hindered β-tubulin binding to CCT. Hence, PDCD5 could interact with CCT to slow β-tubulin folding and disrupt microtubule formation, perhaps in response to apoptotic stimuli (Tracy et al., 2014).

WD40 repeats in proteins consist of multiple 40 amino acid repeats that form a blade of four anti-parallel β-sheets. Typically repeats end with a tryptophan-aspartic acid dipeptide (WD), from which its name is derived. WD40 repeats are widely variable among proteins in terms of sequence and number of repeats, with seven tandem repeats (WD1-7) forming a circular β-propeller being the most common. Early studies revealed that WD40 repeats have different folding kinetics, hence, different chaperone dependencies. CCT, when first identified as interacting with WD40 folds, had the needed shape and features to bind the propeller (Valpuesta et al., 2002). Screening WD40-repeat proteins for CCT interactions showed variable binding that was different from the ordered process of actin folding and suggested a role for CCT in the assembly, in addition to folding, of some WD40-containing proteins (Willison, 2018). One of the WD40 proteins that interact with CCT is the heterotrimeric G protein β subunit (Gβ), an essential component of G protein. Mutational analysis in a cell-free system showed that CCT binding to Gβ was reduced upon deletion of WD40 repeats, most especially upon loss of WD2. Specifically, the β-strand 3 (β3) region of WD2 was essential for CCT recognition, since this region has hydrophobic and polar residues aligned in a “φ-x-φ-x-φ-x” sequence (where φ is a hydrophobic residue) (Kubota et al., 2006). Thus, hydrophobic residues alongside β-strands are likely preferred recognition sites for CCT binding, and subsequent interactions with the chaperonin prevent aggregation and facilitate folding of the native WD40 repeat proteins. This was exemplified in a study of Gβγ dimer formation in which CCT not only folded Gβ subunits but also assisted Gβγ dimer assembly in an ATP-dependent manner (Wells et al., 2006). Cryo-EM studies further revealed that Gβ binds CCT in a near-native state, and subsequent binding with the co-chaperone, PhLP1, helps release Gβ for assembly with Gγ to form the dimer (Plimpton et al., 2015). Like G protein, the anaphase promoting complex/cyclosome (APC/C) is essential for biological activities. APC/C is a ubiquitin ligase that regulates cell cycle phases by targeted protein degradation using as co-activators, the WD repeat-containing proteins, Cdh1 and Cdc20. CCT is the only known chaperone to fold Cdh1 and Cdc20 and interacts directly with WD40 repeats of these proteins, like WD3-5 in Cdc20 (Camasses et al., 2003).

Early studies revealed that the von-Hippel-Lindau (VHL) tumor suppressor is an obligate substrate of CCT (Feldman et al., 1999). VHL is part of the ubiquitin ligase complex that targets proteins like HIF-1α, for degradation. CCT is needed not only for VHL folding but for complexing with partner proteins, elognin B and C. The interaction between VHL and CCT involved hydrophobic residues, with two regions, Box 1 and Box 2, identified as necessary for stable interactions with the chaperonin (Feldman et al., 2003). Crosslinking experiments between CCT and VHL showed that Box1 bound to CCT1 and CCT7 in the H11 of the apical domain region (Spiess et al., 2006). Interestingly, about 50% of the mutations occurring in tumor derived VHL are found in the CCT binding site (Feldman et al., 1999; Feldman et al., 2003). These results highlight the plasticity and specificity of substrate binding to CCT, involving specific configurations of polar and hydrophobic residues in the apical domains of each subunit to accommodate the binding of different substrates.

#### Regulation of Chaperonin Containing TCP1 Subunit Gene Expression

The first CCT subunit discovered was CCT1/TCP-1 (CCTα) encoded by *Ccta*, which is highly expressed in mouse testicular germ cells (Silver et al., 1979). Subsequent studies identified an additional six CCT subunit genes: *Cctb, Cctg, Cctd, Ccte, Cctz*, and *Ccth*, encoding CCT2, CCT3, CCT4, CCT5, CCT6, and CCT7, followed by *Cctq* for CCT8 (Trent et al., 1991; Kubota et al., 1994; Kubota et al., 1995). A ninth subunit gene, *Cctz-*2 expressed in testis was discovered, which distinguished it from *Cctz-1* found in most tissues (Kubota et al., 1997). Current nomenclature for the subunit genes encoding the hetero-oligomeric complex found in most tissues is: *TCP1, CCT2, CCT3, CCT4, CCT5, CCT6a, CCT7*, *and CCT8.* CCT subunit genes have 11–16 exons and contain CpG dinucleotide-rich sequences and SP1 binding sites that are typically found in constitutive housekeeping genes (Kubota et al., 1999b). Using mouse tissues, initial studies of expression found that all eight CCT subunits were distributed across tissues tested, indicating that all eight subunits were required for the activity of the chaperonin. However, expression levels of CCT subunit genes among tissues varied, with the highest expression observed in testis and a mouse mammary tumor cell line. Since these are actively proliferating cells, findings were suggestive that CCT subunit expression correlated with the rate of cell division (Kubota et al., 1999b). CCT expression is also coupled with substrate synthesis Increased CCT expression was observed in testicular germ cells, neuronal cells, and other cells highly dependent on tubulin for synthesis of sperm tails, neurites, or cilia as examples (Kubota, 2002). CCT subunit protein also transiently increased during recovery from chemical stress induced by sodium arsenite, which causes accumulation of unfolded proteins (Yokota et al., 2000b). Searching the first intron of *Ccta* (*CCT1*), a potential binding sequence for heat shock transcription factors (HSF1 and HSF2) was found. Heat shock elements (HSE) for binding HSFs were subsequently located within the first intron or 5’ coding regions of each CCT subunit gene (Kubota et al., 1999a). Since CCT evolved from a common ancestor with other HSPs, the *Cct* genes also have an HSE-HSF transcription system. However, unlike HSPs, upregulation of CCT in response to stress is not always observed. In HeLa cells during heat treatment, HSP70 increased, but not CCT, and a similar outcome was observed in mouse BALB/3T3 cells (Kubota et al., 1999a). CCT also did not increase in response to heat treatments in yeast (Ursic and Culbertson, 1992) or *Tetrahymena* (Soares et al., 1994). Moreover, reducing the levels of CCT did not induce a heat shock response (Grantham et al., 2006). *Cct* genes, having fewer HSEs compared to HSP70, may be less responsive to HSFs and more involved in *de novo* protein folding.

Other transcription factors that regulate the expression of CCT subunit genes include the Staf family members, ZNF143 and ZNF76, that were found in HeLa cells using reporter assays. These bind to two *cis*-acting elements, CAE1 and CAE2, in the promoter for *Ccta* (CCT1 or TCP1) (Kubota et al., 2000). Staf regulates the transcription of small nuclear RNAs (snRNA), snRNA-type genes, and the promoter pol II mRNA (Myslinski et al., 1998); hence Staf family members are essential and their activity in most cells aligns with the fact that CCT1 (TCP-1) is detectable in most tissues. Because of the similarities in CCT subunit expression patterns, ZNF143 and ZNF76 may function as transcriptional regulators in the other seven CCT subunits as well. The transcription of *Cctq* (CCT8) is regulated by a *cis*-acting element comprising 8-base pair long element CCGGAAGT named CQE1 that is bound by the ternary complex factor (TCF): Elk-1, Sap-1a, and Net (Sap-2), which is a subfamily of the Ets domain transcription factors (Yamazaki et al., 2003). Recognition of CQE1 by TCF is independent of serum response factor (SRF). Instead, this transcriptional activity may be regulated by the Ras-mitogen-activated protein kinase (MAPK) pathway since inhibition of MEK (MAPK and ERK Kinase) 1/2 inhibited expression. The RAS/MAPK pathway may play a role in the upregulation of CCT8 after chemically induced stress through activation of the p38 or c-Jun N-terminal kinase/stress activated kinases. Interestingly, inhibition of MEK1/2 also reduced activity from the *Ccta* (CCT1) promoter, thus the MAPK pathway may be a common signaling mechanism to coordinate expression of the CCT subunit genes (Yamazaki et al., 2003).

#### The Activity of Chaperonin Containing TCP1 During Cell Growth

Early observations of CCT expression in cells revealed that the activity of the chaperonin depended on the cell growth rate. Abundant CCT correlated with rapidly dividing cells and CCT decreased during growth arrest or nutrient loss. Re-addition of nutrients or growth factors resulted in recovery of CCT expression in a manner linked with the cell cycle. The cell cycle consists of an initial gap (G1) phase, during which a cell prepares for division, followed by the DNA synthesis (S) phase, a second short gap phase (G2) to assess for DNA damage, and the final phase of mitosis (M) when a cell separates the replicated chromosomes and performs the physical separation into daughter cells during cytokinesis. Initial studies noted that the highest levels of CCT were induced during the G1/S transition and early S phase (Yokota et al., 1999). Moreover, CCT from S-phase arrested cells had more protein-folding activity than CCT from M-phase arrested cells or asynchronously growing cells (Yokota et al., 2001b). In part, these findings could be explained by subunit preferences that correlated with cell cycle phases. For example, CCT1, 4, 6a were increased in S-phase cells compared to M-phase cells. Moreover, measurements of CCT subunit turnover suggested that the rates of synthesis were more variable than the rates of degradation during growth arrest, especially in S-phase arrested cells, enabling subunit levels to increase (Yokota et al., 2001b; Kubota, 2002).

One reason for the heightened need of CCT during the cell cycle is the critical role that both tubulin and actin play during cell division. During M phase, the formation of the mitotic spindle is essential for the segregation of replicated chromosomes. Microtubules formed by tubulin are the building blocks of the mitotic spindle. During cytokinesis, when dividing cells physically separate, the contractile ring that forms will contain F-actin along with myosin motors. Providing functional forms of these cytoskeletal proteins is a key function of CCT during the cell cycle. Early studies showed that high levels of tubulin were associated with S-phase CCT complexes, especially during the G1-S transition, but not in G1-arrested cells (Yokota et al., 1999). Targeting the apical domain of CCT5 with a microinjected antibody or using small interfering RNAs (siRNAs) to inhibit individual CCT subunits in 3T3 cells caused a delay of the G1-S transition and the accumulation of disordered microtubules (Grantham et al., 2006; Brackley and Grantham, 2010). Hence, the depletion of CCT subunits reduced tubulin levels and impacted actin folding, showing the important relationship between substrates like actin or tubulin and CCT during cell growth.

Control of the cell cycle in eukaryotic cells is driven by cyclins and cyclin-dependent kinases (CDKs). CDKs are inactive until bound by their cyclin partners, forming CDK-cyclin pairs that function in specific phases of the cell cycle. CDKs are subject to regulatory phosphorylations/dephosphorylations that fine tune their activity to drive cell cycle progression. The CDK2-cyclin E complex is active from late G1 through S-phase. Using yeast cells, in which overexpression of cyclin E and CDK2 is lethal, a genetic screen of negative regulators of cyclin E revealed that expression of human CCT2, CCT3, and CCT8 rescued the lethality caused by cyclin E expression, perhaps acting as dominant negative subunits in this mix of yeast and human proteins. Further work showed that CCT transiently associated with newly translated cyclin E, and that ATP was required for release from the chaperonin, which was confirmed by co-immunoprecipitation of CCT with cyclin E in the presence of EDTA to inhibit ATP and magnesium (Mg). Using HeLa cells arrested in S-phase, the ATP-dependent physical interaction between cyclin E and CCT was shown (Won et al., 1998). However, subsequent studies found that the folding of cyclin E by CCT was not required for CDK2 binding, and that the association of cyclin E with the chaperonin was processed distinctly from obligate substrates like actin, suggesting that the interaction of CCT with cyclin E may be indirect (Grantham et al., 2006). While early work found that CDK2 did not precipitate in complex with CCT (Won et al., 1998), a mass spectrometry study of the cellular processes involving CDK2 identified multiple CCT subunits as physical interactors of CDK2 (Neganova et al., 2011). In fact, data from the protein interactors database, BioGRID (https://thebiogrid.org), indicates that CCT subunits can interact with hundreds of proteins, suggesting that many proteins may occupy CCT complexes at a low abundance. As example, a proteomic screen for G1 phase proteins identified multiple CCT subunits as interactors of cyclin D1 (Jirawatnotai et al., 2011).

Progression through the cell cycle leads to M phase during which nuclear division takes place. M phase is highly coordinated to ensure that duplicated chromosomes are equally distributed into the pair of daughter cells. Sister chromatids attach to the mitotic spindle in early M phase and separation and segregation of sister chromatids to opposite ends of the cell occur in the later stages of M phase. Critical to the latter process is the degradation of the cell cycle regulator, securin, to unleash separase that triggers the separation of chromatids from the mitotic spindle. Mitotic protein degradation is mediated by the APC/C complex that is activated by binding the co-activator, Cdc20. Checkpoint proteins, Mad1, 2, 3 and Bub1, 3 control the spindle checkpoint by inhibiting Cdc20 (and proteolysis) until all chromatids attach to the spindle. Layered onto this regulation is the role of CCT in folding the functional form of Cdc20. Studies in budding yeast showed that CCT subunits co-migrated with Cdc20 in a glycerol density gradient, and immunoprecipitants revealed stable interactions between CCT subunits and Cdc20, but no other components of the APC/C complex; hence, the binding between CCT and Cdc20 is specific. Mutations in CCT subunits abolished the interaction with Cdc20, suggesting that a functional chaperonin was required. Further, the interaction of CCT with Cdc20 was ATP-dependent, since non-hydrolyzable analogs of ATP inhibited binding. Cdc20 has three regions: the N-terminal domain that has the Mad2 binding sites, seven WD40 repeats, and a short C-terminal region. Deletion studies established that the WD40 repeats were the sites of interaction with CCT. Together this body of data indicates that CCT is required to produce a functional APC/C-Cdc20 complex needed to exit the spindle checkpoint in M phase. CCT is also required to produce functional Cdh1, the G1-specific co-activator of APC/C that ensures the completion of M phase and entry into G1 through destruction of mitotic cyclins (Camasses et al., 2003).

Polo-like kinases (Plk) are important components of the cell cycle, specifically active during the transition to M phase, the regulation of mitotic exit, and cytokinesis. Plk1 supports the maturation of the centrosome and spindle assembly, phosphorylating among its targets the Cdc25 family of phosphatases that remove inhibitory phosphorylations from CDK-cyclins. Using a yeast two-hybrid screen, Plk1 was identified as a possible interactor of CCT, which was confirmed *in vitro* using a rabbit reticulocyte lysate (RRL) system by co-immunoprecipitating Plk1 with CCT1, and *in vivo* using mouse mammary gland carcinoma FT210 cells. Depleting CCT1 by RNA interference (RNAi) in HeLa cells caused an arrest in G2/M phase, preventing mitotic entry. Continued depletion of CCT1 later resulted in apoptotic cell death, which could be prevented by inducing a G1 arrest to keep cells from entering M phase. Decreasing CCT1 also reduced levels of active Plk1 and resulted in low CDK activity that prevented progression through M phase. The addition of active Plk1 or cdc20 reversed the effects of CCT1 loss. Depletion of Plk1 recapitulated the G2-M arrest phenotype observed with depletion of CCT1 (Liu et al., 2005). Hence, Plk1 is likely a substrate of CCT. HSP90 was also found to regulate the stability of Plk1 (Simizu and Osada, 2000). Nascent Plk1 may associate with CCT co-translationally and then reach its functional form with the help of HSP90.

A novel function of CCT in the eukaryotic cell cycle is its role in the disassembly of the mitotic checkpoint complexes (MCC), which is distinct from the protein folding activity described for Cdc20 or Plk1. The MCC is formed by the checkpoint proteins: Mad2, BubR1, Bub3, and Cdc20. Studying the pathways of MCC disassembly led to the discovery of an ATP-dependent factor that enabled the release of Cdc20 from the MCC. This factor was identified as CCT and confirmed by generating CCT5 oligomers that could release Cdc20 (and Mad2) from the MCC, albeit with reduced activity compared to purified hetero-oligomeric CCT. Like its protein-folding activity, MCC disassembly by CCT was dependent on ATP hydrolysis (Kaisari et al., 2017). How this mechanism differs from the chaperonin’s protein-folding activity, especially since CCT also folds Cdc20, is unknown, but could involve interactions with the WD40 repeats of Cdc20 and employ the chaperonin’s capacity to sequentially bind domains of complex proteins in its central chamber.

Regulation of CCT protein-folding capacity could be achieved through PTMs as described for HSP70 and HSP90. For example, HSP70s are highly phosphorylated, especially during cell cycle progression, and their activity can be regulated by the MAPK pathway [reviewed in (Nitika et al., 2020)]. Likewise, in a study of CCT activity during growth factor signaling, CCT2 was phosphorylated by p90 ribosomal S6 kinase (RSK) and p70 ribosomal S6 kinase (S6K), serine/threonine kinases that are activated by the MAPK pathway and/or the phosphoinositide 3-kinase (PI3K)-mammalian target of rapamycin (mTOR) pathway, respectively. Importantly, depletion of CCT2 by RNAi inhibited cell proliferation, and complementation with an ectopically expressed CCT2 S260D mutant (phosphomimetic) restored cell growth, but not with a CCT2 S260A mutant that was phosphodeficient, indicating that Ser-260 phosphorylation may modulate the protein-folding activity of CCT during cell growth and possibly in the context of oncogenic stimulation (Abe et al., 2009).

### Chaperonin Containing TCP1 as a Preventive or Causative Factor in Disease

#### Chaperonin Containing TCP1 in Neurological Disorders: Defects in Protein Folding and Aggregation

Aberrant protein folding and protein aggregation often underlie the development of neurological disorders. The onset of Parkinson’s disease, Alzheimer’s disease, or polyQ expansion diseases is associated with the accumulation of misfolded proteins in cells. In Huntington’s disease, the expansion of polyQ tracts in huntingtin (Htt) protein is a hallmark of disease (DiFiglia et al., 1995). Mutant Htt (mHtt) containing greater than 40 polyQ tracts can misfold and aggregate, causing toxicity and neural damage through mechanisms still being studied. In a genome wide screen for genes that could regulate polyQ aggregation, a number of genes involved in protein folding were identified, including HSP70, CCT5, and five other CCT subunits (Nollen et al., 2004). Subsequent work in a yeast model with an aggregating form of Htt, Htt53Q, showed that the CCT hetero-oligomer could reduce fibril elongation and function cooperatively with HSP70 to inhibit the fibrillar aggregation of polyQ expansion proteins and suppress toxicity (Behrends et al., 2006). Disruption of CCT in HEK293 cells expressing polyQ-EYFP or Htt-fusion constructs, by depleting CCT6 using RNAi, caused polyQ-expansion-related cell death and a twofold increase in the aggregation of polyQ proteins; while overexpression of all eight CCT subunits (but not single subunits) in Htt-expressing Neuro2a cells reduced polyQ aggregation (Kitamura et al., 2006). In a subsequent study, overexpressing CCT1 or CCT4, or just the apical domain of CCT1 (ApiCCT1) in Htt-expressing yeast cells or mammalian cells was sufficient to change the morphology of Htt aggregates, indicative of a possible mechanism for the suppressive behavior of CCT that was distinct from the protein-folding activity of the hetero-oligomer (Tam et al., 2006; Tam et al., 2009; Sontag et al., 2013) (Table 1).

**TABLE 1:** CCT involvement in Neurological Disorders and other diseases.

Neurological diseases	CCT subunits	Mechanism of action	Citations
Alzheimer’s Disease	CCT2	Downregulated in patients	Liu et al. (2020)
Axonal transport	CCT3, CCT1 (apical)	Individual subunits normalized axonal transport and lysosomal transport	Zhao et al. (2016)
	CCT5	Regulated CDK5/p35 to increase phospho-Tau	Chen et al. (2018)
Huntington’s disease	CCT1	Single subunit reduced Htt-induced toxicity	Tam et al. (2006)
	CCT complex, CCT1	Suppressed mHtt aggregation	Tam et al. (2009)
	CCT1 (apical domain)	Inhibited aggregation of mHtt and reduced mHtt-toxicity	Sontag et al. (2013)
	CCT complex	Capped mHtt fibril tips and encapsulated smaller mHtt oligomers	Shahmoradian et al. (2013)
	CCT5	Homo-oligomer inhibited mHtt aggregation	Darrow et al. (2015)
	CCT3, CCT1 (apical)	Individual subunits reduced mHtt	Zhao et al. (2016)
Neuropathy	CCT5	Missense A492G mutation causing His147Arg described	Bouhouche et al. (2006)
	CCT5	His147Arg mutation examined in archaeal homolog	Min et al. (2014)
	CCT5	Biochemical analysis of His147Arg mutants	Sergeeva et al. (2014)
	CCT5	Structure of His147Arg mutation resolved	Pereira et al. (2017)
	CCT5	Leu224Val mutation described	Antona et al. (2020)
Parkinson’s disease	CCT2	Subunit upregulated in MPP + -treated SH-SY5Y cells	Xie et al. (2016a)
	CCT7	Oxidative stress-induced neuronal apoptosis	Anantharam et al. (2007)
	CCT complex, CCT3/6	Interfered with amyloid fibril assembly	Sot et al. (2017)
PolyQ expansion proteins	CCT complex, CCT5	Genome-wide RNA interference screen for PolyQ aggregation suppressors	Nollen et al. (2004)
	CCT complex	Synergistic function with HSP70 suppressed polyQ toxicity	Behrends et al. (2006)
	CCT complex	CCT6 knockdown and CCT1-8 overexpression modulated polyQ folding	Kitamura et al. (2006)
	CCT1	Single subunit reduced Htt-induced toxicity	Tam et al. (2006)
	CCT complex, CCT4	VRK2-mediated downregulation of CCT4 and polyQ aggregation	Kim et al. (2014)
	Targeted CCT2/5/7	Loss of function inhibited autophagy, causing protein aggregation	Pavel et al. (2016)
Protein aggregates	CCT complex, CCT4	USP25 catalyzed the de-ubiquitination of CCT reducing polyQ aggregation	Kim et al. (2015)
**Other diseases**	**CCT Subunits**	**Mechanism of action**	**Citations**
Amyotrophic lateral sclerosis	CCT5, CCT7, CCT8	Overexpressed in cytoplasm of mutant cells	Kim et al. (2017)
Primary biliary cirrhosis	CCT5	Upregulated compared to normal tissue, decreased with treatment	Chen et al. (2008)
Diabetes	CCT8	Increased in insulin-resistant vs. insulin- sensitive	Xie et al. (2016b)
	Complex, CCT8, CCT4	Increased in insulin resistance	Hwang et al. (2010)
Down syndrome	CCT5	Decreased in parietal cortex	Yoo et al. (2001)
Epilepsy	CCT1	Altered protein in hippocampi	Yang et al. (2006)
Atrial fibrillation	CCT5	Elevated protein in atrial tissue	Goudarzi et al. (2011)
Coronary artery disease	CCT6A	Upregulated gene expression based on microarray data	Balashanmugam et al. (2019)
Hepatitis C virus	CCCT complex, CCT5	Role in virus replication	Inoue et al. (2011)
Rabies virus	TCP1	Role in virus replication	Zhang et al. (2014)
*Clostridium difficile* Toxins	CCT4/5	Directly interacted with toxins	Steinemann et al. (2018)
Pain	CCT5	Increased expression levels in pain models	Peters et al. (2013)

Structural studies showed that the CCT hetero-oligomer can sequester a short Htt sequence responsible for aggregation and in this manner reduce mHtt aggregation (Tam et al., 2009). Using cryo-EM microscopy and cryo-electron tomography, the 3D structure of CCT with mHtt containing an expanded polyQ tract with 51 residues (mHtt51) was resolved. CCT capped the fibril tips of mHttQ51 through the apical domains of its subunits as well as encapsulated smaller mHtt oligomers within its central cavity. Together these mechanisms are proposed to inhibit the *in vitro* aggregation of mHtt (Shahmoradian et al., 2013). In addition, a homo-oligomer composed of CCT5 could also cap fibrils and encapsulate mHtt oligomers, as shown using cryo-EM tomography, suggesting a shared mechanism with the CCT hetero-oligomer (Darrow et al., 2015). While these studies explain why CCT depletion enhances polyQ aggregation, the actual mechanism may be more complicated. In addition to protein folding, CCT supports lysosomal activity, so its loss could impair the process of autophagy. CCT also modulates the assembly of the mechanistic target of the mTOR complex, which is a regulator of autophagy (Cuellar et al., 2019). Using HeLa cells and mouse cortical neurons, CCT2/5/7 subunits were depleted by RNAi, which reduced autophagosome formation due to the defective folding of actin and increased polyQ aggregation. Importantly, when CCT was depleted in autophagy-deficient HeLa cells, the aggregation of polyQ proteins did not change. Hence, CCT loss resulted in an autophagy deficiency that caused the accumulation and aggregation of disease-causing proteins like mHtt (Pavel et al., 2016). Hence both direct effects of CCT on polyQ expanded proteins and indirect effects through cellular processes, like autophagy, contribute to the activity of CCT in reducing protein aggregate formation.

To determine if CCT controls the aggregation of other proteins, the action of the chaperonin on the amyloid fibril assembly of α-synuclein (α-syn) with the A53T mutation was examined. α-syn is a key protein involved in the pathogenesis of Parkinson’s disease. CCT interfered with fibril assembly through specific interactions of CCT3 and CCT6, as part of the hetero-oligomeric complex, with the α-syn A53T central hydrophobic region. Since CCT partially encapsulated the A53T oligomers, closure of the ring induced by ATP binding and hydrolysis did not occur; hence, incomplete encapsulation is suggestive of ATP-independent inhibition of fibril assembly (Sot et al., 2017). Since CCT also inhibits mHtt aggregation in a similar nucleotide-independent manner (ApiCCT1), this could be indicative of a passive mechanism that reduces neurotoxicity by sequestering toxic proteins, as well as improving axonal transport of brain-derived neurotrophic factor (BDNF) needed for neurons (Zhao et al., 2016). Alternatively, *in vivo,* the cooperation of co-chaperones, like HSP70, may be needed to help CCT fully resolve misfolded proteins that cause neurological disorders.

Mutations in CCT subunits are associated with neuropathies (Table 1). In a rare case of mutilating hereditary sensory neuropathy, a missense mutation on exon four of *cct5* resulted in the substitution of a conserved histidine for arginine at amino acid 147 (H147R) (Bouhouche et al., 2006). This mutation is in the equatorial domain of CCT5 near the region where ATP/ADP binds. Using an archaeal Cpn60 homolog that is similar to CCT5, the reduction in ATP activity and defective oligomerization capacity were attributed to the histidine to arginine mutation in CCT5, demonstrating for the first time how a gene defect in CCT can cause disease (Min et al., 2014). Folding assays using γD-crystallin and actin substrates demonstrated the reduced folding activity of the H147R protein, but complete folding activity was not lost (Sergeeva et al., 2014). The subsequent crystal structure of CCT5 H147R mutant revealed that the position of R147 in CCT5 should not impair ring interactions or prevent assembly of the complex, nor block ATP binding. However, contacts between the side chains of R147 with other residues could impact the flexibility of the equatorial domain, changing the cooperativity within the rings of the complex that could reduce activity (Pereira et al., 2017). The importance of the CCT5 subunit in preventing neurological disorders was further shown in a different case of early-onset demyelinating neuropathy in which a L224V mutation in the intermediate domain of CCT5 was described. Similar modeling of CCT5 L224V, with or without nucleotides, showed that the apical domain was the most changed, affecting the subunit’s conformation (Antona et al., 2020). Aberrant expression of other CCT subunits, like CCT2, also correlated with Alzheimer’s disease or Parkinson’s disease and could be used as biomarkers to assist with neurological disease prognosis and management (Brehme et al., 2014; Xie H. et al., 2016; Liu et al., 2020).

#### Chaperonin Containing TCP1 in Cancer: Increased Protein Folding Activity

Beginning with the first report of CCT in hepatocellular carcinoma (HCC) and colon cancer (Yokota et al., 2001a), the increased expression of individual CCT subunit RNA and protein in tumor tissues compared to normal tissues was shown (Table 2). Consistent with this body of data is that CCT expression increased with tumor grade—the more advanced the tumor the higher the levels of CCT—and correlated with poor patient outcomes or prognosis. In recent years, the increasing amount of patient data available in datasets (e.g., The Cancer Genome Atlas (TCGA), The Human Protein Atlas, Oncomine etc.), provide strong support for CCT as a driver of cancer and potentially an oncogene (Klimczak et al., 2019; Dong et al., 2020; Liu et al., 2020). In many cancers, the genes encoding CCT subunits are genomically amplified and found in chromosomal hotspots (e.g., *CCT2*). Unlike the neurological disorders, loss of function CCT gene mutations are rarely seen in cancer (Ghozlan et al., 2021). Depletion studies of individual CCT subunits demonstrated the essential role of the chaperonin in cancer cell proliferation, cancer cell invasion and migration, and cancer cell death, advancing research on the chaperonin as a potential prognostic marker and therapeutic target in the management of cancer (Table 2).

**TABLE 2:** CCT subunits are highly expressed in different cancers.

Cancers	CCT subunits	Mechanism of action	Citations
Adenocarcinomas	CCT2	Positive expression in tumor tissue correlated with clinical behavior	Zou et al. (2013)
Breast cancer	CCT2, TCP1	Genes identified as being recurrently altered and necessary for growth of cancer cells	Guest et al. (2015)
	CCT2	Gene and protein expressed in tumor tissues that correlated with patient outcomes and identified as a targeted peptide therapeutic	Bassiouni et al. (2016)
	CCT6A	RNA and protein were increased in tumor tissues compared to non-tumor tissues and associated with poor patient survival	Huang et al. (2019)
	CCT1, CCT2, CCT6A	Bioinformatics analysis revealed overexpression correlated with unfavorable prognosis with implications for other cancers	Klimczak et al. (2019)
	CCT2	Gene increased in patients and correlated with poor survival; depletion reduced tumor growth in mice	Showalter et al. (2020)
	CCT2, 3, 4, 5, 6A, and 7	Extensive data mining revealed that subunits were increased in tumor tissue compared to nontumor tissue and correlated with immune cell markers	Xu et al. (2021)
	CCT2	Genomically amplified in cancers that correlated with reduced patient survival and co-occurrence reported with genes that regulate the cell cycle	Ghozlan et al. (2021)
	CCT2	Transcriptomic profiling found increased expression in multiple cancers; association with PR+, HER2-, and advanced tumor stage; had prognostic value for luminal A cancers	Liu et al. (2021b)
	CCT2	Bioinformatics and patient data correlated increased expression with patient prognosis; developed as a biomarker for diagnostic assay to detect circulating tumor cells	Cox et al. (2022)
Colorectal cancer	CCT2, CCT5	Protein detected in tumor tissues that correlated with advanced stage and poor patient survival	Coghlin et al. (2006)
	cirCCT3	Circulating RNA was highly expressed in clinical tumors	Li et al. (2020)
	CCT2	Tumor tissues showed higher expression than normal colon tissue that correlated with reduced patient survival	Park et al. (2020)
Esophageal squamous cell carcinoma	CCT8	Protein expression was high in tissues from patients with lymph nodes metastasis and overall survival was poor	Yang et al. (2018)
Ewing sarcoma	CCT6A	Bioinformatics analysis correlated high expression with poor prognosis; possible biomarker	Jiang et al. (2021)
Gastric cancer	CCT3	High levels detected in cancer tissue compared to adjacent healthy tissue and knockdown reduced growth of cancer cells *in vitro* and *in vivo*	Li et al. (2017)
Glioma	CCT8	Protein detected in tumor tissue and cell lines correlated with tumor grade; knockdown reduced growth and migration	Qiu et al. (2015)
Glioblastoma	CCT6A (also: CCT2, 3, 5, TCP1)	Neurosurgical aspirates contained extracellular vesicles with detectable protein that negatively correlated with patient survival	Hallal et al. (2019)
Head and neck squamous cell	CCT complex, CCT4, CCT7	Database mining showed that gene expression was higher in tumors than normal tissues and correlated with low patient survival and advanced stage	Dong et al. (2020)
Hepatocellular and colonic carcinoma	CCT Complex, CCT2	Increased expression in tumor tissue compared to non-tumor tissue and correlated with cell growth indicators	Yokota et al. (2001a)
Hepatocellular carcinoma	CCT8	High expression correlated with histological grade and tumor size; also associated with poor prognosis	Huang et al. (2014)
	CCT3	Increased expression in cell lines and tissue	Qiu et al. (2015)
	CCT Complex	Using patient samples and database mining, increased expression correlated with poor prognosis and dysregulated oncogenes	Yao et al. (2019)
	CCT5, CCT complex	Dataset mining found that RNA and protein are upregulated in tumors and associated with advanced tumor grade and poor survival; depletion or overexpression altered growth, invasion	Liu et al. (2021a)
	CCT6A	RNA and protein were increased in tumor tissues and associated with poor survival; depletion in cells reduced proliferation	Zeng et al. (2019)
	CCT1 (TCP1)	High levels observed in poorly differentiated tissue that was increased over normal adjacent tissues, which correlated with shorter survival and worse prognosis	Tang et al. (2020)
	CCT7	Dataset mining showed that gene was higher in cancer tissue compared to normal and RNA and protein were independent risk factors for poor prognosis	Huang et al. (2022)
Multiple myeloma	CCT3	Database mining correlated expression with poor prognosis; potential role in diagnosis	Qian et al. (2020)
Non-small cell lung cancer	CCT5	Proteomics analysis showed that protein was detectable in sera (autoantibody response) and was expressed in tissues	Gao et al. (2017)
	CCT6A	Increased expression in tumor tissues compared to tumor adjacent, associated with lymph node metastasis and negatively correlated with patient outcomes	Zhang et al. (2020)
Osteosarcoma	CCT complex, CCT4	Database mining showed increased expression, with the highest among most cancers; expression correlated with metastasis and poor survival; small molecule inhibitor tested	Wang et al. (2022)
Pancreatic cancer	CCT8	Mass spectrometry analysis detected protein in invasive cell line that could be secreted in exosomes; potential use in liquid biopsies	Liu et al. (2019a)
Prostate cancer	CCT2	Protein increased in cell lines that were susceptible to targeted therapeutic	Flores et al. (2017)
Small cell lung cancer	CCT2	Protein highly expressed in tumor compared to normal lung and correlated with increasing grade and poor survival; targeted therapeutic reduced growth in cell lines	Carr et al. (2017)
Thyroid cancer	CCT3	Protein was increased in tumor tissues compared to matched controls and knockdown reduced cell proliferation	Shi et al. (2018)

Early studies showed that CCT activity is necessary for cancer cell proliferation due to the need for the functional forms of cytoskeletal proteins as well as cell cycle regulators like Cdc20 and Cdh1 (Grantham et al., 2006). Cancer cell lines tend to express more CCT than non-cancer cells, although expression and activity may not always correlate (Boudiaf-Benmammar et al., 2013; Bassiouni et al., 2016). Depletion of CCT2 in triple-negative breast cancer (TNBC) cells prevented tumor growth in a murine syngeneic model (Showalter et al., 2020) and impaired the formation of tumor spheroids (Ghozlan et al., 2021). Knockdown of CCT3 in a mouse model of gastric cancer reduced tumor size (Li et al., 2017). Depletion of CCT3 in thyroid gland papillary carcinoma cells, TNBC cells, or gastric cancer cells decreased cell cycle progression and caused cell cycle arrest, altering signal transduction pathways that drive cell cycling (Li et al., 2017; Shi et al., 2018; Xu et al., 2020). Similarly, pathway analysis positively correlated CCT6A with G2-M phase cyclins B and A in breast cancer (Huang et al., 2019). Knockdown of CCT8 in esophageal squamous cell carcinoma (ESCC) cells downregulated actin and tubulin and enhanced cell death upon treatment with cisplatin (Yang et al., 2018). CCT8 depletion in glioblastoma multiforme (GBM) cells affected the cytoskeleton and decreased proliferation and invasion, which was also observed in HCC cell lines (Huang et al., 2014; Qiu et al., 2015). While such studies provide additional evidence for an essential role of CCT in cancer cell growth, it is unclear from CCT subunit depletion experiments if effects are monomer specific, as was suggested for neurological disorders, or dependent on the depleted subunit for CCT complex assembly. In many of these studies, loss of cytoskeleton proteins was observed, suggestive of the latter idea—that the CCT hetero-oligomeric complex is needed to support cancer cell cycling. The consequence of depleting one CCT subunit on the rest of the subunits is not fully understood; yet evidence suggests that the remaining subunits may also decrease (Showalter et al., 2020). Hence, more work is needed to determine if there are unique activities for CCT subunit monomers in cancer cells.

Profiling more than 9,000 tumors from the TCGA revealed that most cancers have genetic alterations in 10 major signaling pathways (cell cycle, p53, MYC, PI-3 kinase/AKT, JAK-STAT, Hippo, Wnt, and TGF-beta among others) (Sanchez-Vega et al., 2018). Using the BioGRID database to search for protein interactions involving CCT revealed that there are potential CCT subunit interactors in all these signaling pathways that drive cancer (Ghozlan et al., 2021), supporting that CCT may promote oncogenesis through mechanisms that go beyond producing the functional forms of actin and tubulin.

The folding of the transcription factor, p53, is mediated by interactions with CCT. p53 is a tumor suppressor that prevents cancer development by activating pathways inducing cell cycle arrest or apoptosis when DNA damage is detected. In cancer, p53 is often mutated or ablated. Tumor-associated mutations can result in the generation of misfolded p53 that is unstable, which results in loss of the protective wild-type activity and a gain-of-function activity that promotes cancer invasiveness. CCT subunits were identified as part of the p53 interactome (Coffill et al., 2012) and immunoprecipitants of CCT1 (TCP-1) or CCT5 with wild-type or mutant forms of p53 were detected in cancer cells overexpressing the p53 constructs (Trinidad et al., 2013). Depletion of CCT subunits resulted in some loss of p53 binding to the chaperonin complex, but since other chaperones, including HSP70, can also fold p53, the existence of a cooperative mechanism for p53 folding cannot be ruled out. CCT binds to an N-terminal region of p53 that may involve hydrophobic interactions, since treatment with chaotropic salts disrupted binding. Depletion of CCT also decreased invasion in p53 mutant-expressing cancer cells, but since CCT folds other proteins (like actin) this effect may not solely be due to reduced folding of the p53 mutant proteins. However, depletion of both p53 and CCT decreased the invasive capacity of cancer cells (Trinidad et al., 2013). Interestingly, the negative regulator of p53, MDM2, may also be a CCT interacting protein, since it was identified in a mass spectrometry analysis of proteins in cancer cells captured by a FLAG-MDM2 construct (Yamauchi et al., 2014). MDM2 is also in the same chromosomal amplicon as CCT2 that is highly expressed in cancer cells (Ghozlan et al., 2021). While CCT may contribute to the correct folding of wild type p53, how its increased expression in cancer cells supports the mutant or unfolded forms of p53 remains to be determined but could involve interactions with other proteins like MDM2 or PTMs that modify CCT activity. As an example, functional enrichment analysis or treatments with inhibitors suggested that the function of CCT could be regulated through the PI3 kinase/AKT pathway (Guest et al., 2015; Dong et al., 2020). In fact, gene set enrichment analysis of mutant p53 identified a gene signature highly enriched in targets of the MYC transcription factor that were downregulated in head and neck squamous cell carcinoma (HNSCC) by treatment with a PI3 kinase inhibitor. Importantly, CCT2 was one of the MYC target genes, and mutant p53, MYC, and YAP bind to the promoter of CCT2, regulating its expression (Ganci et al., 2020). Whether CCT2 is part of a feedback loop that could promote the invasiveness of mutant p53-expressing cancer cells is speculation that will require further investigation to determine.

A signaling pathway often perturbed in cancer cells is the Janus tyrosine kinase (JAK)-signal transducers and activators of transcription (STAT). Among JAK-STAT members, STAT3 is linked to the enhancement of cancer cell proliferation, migration, and suppression of anti-tumor immune responses (Yu et al., 2014). CCT was found to bind to STAT3. CCT2, and CCT5 were immunoprecipitated with newly transcribed STAT3 in RRLs and knockdown of CCT2 (which also reduced other subunits) decreased the levels of STAT3 in epithelial cell lines. Using a mixed RRLs approach in which STAT3 was co-expressed with single CCT subunits, STAT3 bound mainly to CCT3, likely through its DNA-binding domain (Kasembeli et al., 2014). Constitutively activated STAT3 promotes the oncogenic process through the expression of genes like *CCND1* (cyclin D1), a key driver of cell cycle progression when partnered with its cognate CDK (Leslie et al., 2006). CCT3 expression in multiple myeloma was associated with signaling pathways that included JAK-STAT3. The set of genes upregulated in the CCT3^high^ group of patients correlated with those resulting from IL-6/JAK/STAT3 stimulation (Qian et al., 2020). CCT itself could activate genes that drive cell cycling, since overexpression of CCT2 in luminal A breast cancer cells increased the gene expression of *MYC* and *CCND1* that statistically correlated with *CCT2* (Ghozlan et al., 2021), while depletion of CCT6A in an HCC cell line decreased levels of cyclin D (Huang et al., 2019). Amplified CCT expression in cancer cells could, therefore, directly promote cell cycling by folding essential substrates like tubulin or cdc20 and indirectly through interactions with transcription factors like MYC, and promoting the expression of cell cycling factors like cyclin D.

Focusing on cancers in which CCT subunits were overexpressed revealed more ways that amplification of CCT could promote oncogenesis. Using two cancer cell lines [triple negative breast cancer (TNBC) and non-small cell lung cancer (NSCLC)] that highly expressed CCT2, depletion of this subunit decreased anti-apoptotic proteins like XIAP and inhibited phosphorylation of AKT and GSK3. Overexpression of CCT2 in CCT2-depleted cells restored the AKT-GSK3β-β-catenin and XIAP-survivin pathways, showing that the CCT complex may directly bind and stabilize XIAP and β-catenin (Chang et al., 2020). CCT3 supported breast cancer cell proliferation through the nuclear accumulation of β-catenin, perhaps directly since signals from CCT3 and β-catenin co-localized in the nucleus. A connection between CCT3 and microRNA (miR) MiR-223 further supports that CCT3 regulates the expression of β-catenin and therefore is involved in the competing endogenous RNA (ceRNA) network that regulates other genes like STAT5 through miR-223 (Qu et al., 2020). Depletion of CCT1 in HCC cells produced a similar outcome with reduction of Wnt signaling molecules Wnt7b and β-catenin (Tang et al., 2020), while in colorectal cancers (CRC), the presence of circulating RNA for CCT3 (circCCT3) was linked to advanced CRC and stimulated vascular growth endothelial factor A (VEGFA) and the Wnt signaling pathway (WNT3). CRC can also be promoted through hypoxia-driven signaling due to the loss of oxygen in these solid tumors. Hypoxic conditions activate the Hedgehog pathway. Gli-1, a Hedgehog signaling factor, interacted with CCT2, shown by mass spectrometry analysis of CRC cells. Depletion of CCT in CRC cells caused ubiquitination and degradation of Gli-1, likely through misfolding. Hence, in this model, the inhibition of CCT2 prevented tumor growth through decreases in Gli-1 levels (Park et al., 2020). In liver cancer, CCT3 was elevated and co-interacted with the transcriptional co-activator YAP, which is negatively regulated by the Hippo pathway, and transcription factor CP2 (TFCP2), a Hippo-independent oncoprotein in liver cancer. The pro-tumorigenic activity of CCT3 increased the half-lives of YAP and TFCP2 by preventing their interaction with the ubiquitination machinery (Liu Y. et al., 2019). CCT is also involved in TGF-β signaling. CCT6A bound SMAD2, a signal transducer of the TGF-β pathway, and suppressed SMAD2-mediated gene transcription in NSCLC cells, inducing metastasis in an animal model when overexpressed (Ying et al., 2017). While most of the known pro-cancer activity of CCT is in solid tumors, a role for CCT in acute myeloid leukemia was described. AML1-ETO is the result of a translocation in which two genes generate a fusion oncoprotein. The synthesis and folding of AML1-ETO are done by CCT, with the help of HSP70, by interacting with the fusion protein’s β-strand rich, DNA-binding domain. Interestingly, the binding of AML1-ETO with CCT could occur under nucleotide-free conditions, which differs from the ATP-dependent folding model (Roh et al., 2016). This may be an example of a mechanism involving the apical domains of CCT subunits, in which CCT, like GroEL, could function as an “unfoldase” (Priya et al., 2013).

#### Chaperonin Containing TCP1: Diagnostics and Therapeutics

One of the first CCT modulating compounds was Heat Shock Factor 1A (HSF1A), identified in a humanized yeast-based high-throughput screen for small molecule activators of HSF1. HSF1A reduced polyQ aggregation in neuronal precursor cells, which decreased cellular toxicity. But instead of binding to HSP90, HSF1A interacted with CCT1/TCP-1 and CCT8, shown in a biotin-based pull-down experiment, to regulate the activity of the chaperonin (Neef et al., 2010). A more specific interaction was targeted with the compound I-Trp, an iodomethyl ketone warhead that alkylates Cys^354^ of β-tubulin. I-Trp disrupted protein-protein interactions between the CCT and tubulin, specifically targeting cancers that overexpress CCT2 (Lin et al., 2009; Liu et al., 2017). Specific interactions involving CCT4 were targeted with another small molecule compound called anticarin-β, which displayed more selectivity for cancer cells than normal cells. Anticarin-β is a natural coumarin compound from *Antiaris toxicaria* Lesch that inhibited the CCT4-mediated maturation of STAT3, among other effects, such as inhibiting the lysosomal-autophagy pathway. Anticarin-β reduced tumor growth in orthotopic and patient-derived xenograft models of osteosarcoma (Wang et al., 2022). A different approach to CCT inhibition was based on a peptide therapeutic derived from the apoptotic protein, Bax, called CT20p (Boohaker et al., 2012). CT20p displayed selective anti-cancer toxicity that was caspase-independent, and death involved disruption of the cytoskeleton (Lee et al., 2014). In a proteomics-based pull-down experiment with biotinylated CT20p, seven of eight CCT subunits were identified as interactors of the peptide. CT20p directly bound to recombinant CCT2 in a cell-free system as well as to cytosolic CCT2 in an “in cell” pull-down assay. CCT2-depleted cancer cells were resistant to killing with CT20p, while cancer cells highly expressing CCT2 were sensitive (Bassiouni et al., 2016). Using polymeric nanoparticles to systemically deliver CT20p to cancer cells resulted in tumor growth inhibition in prostate and breast cancer animal models and small cell lung cancer cells (Lee et al., 2014; Carr et al., 2017; Flores et al., 2017). How CT20p inhibits CCT could be in line with the amphipathic nature of the peptide comprised of hydrophilic and hydrophobic amino acids (Boohaker et al., 2012).

Developing diagnostics targeting CCT subunits takes advantage of novel methodologies such as liquid biopsy using blood or serum (Tables 1, 2). In Alzheimer’s disease, CCT2 was part of a gene set used to develop a diagnostic profile (Liu et al., 2020). Likewise in breast cancer, CCT1 and CCT2 were part of HSP signature that could predict outcomes and help stratify patients for treatment (Klimczak et al., 2019). In pancreatic cancer, CCT8 was secreted from an invasive pancreatic cancer cell line, likely through the exosome pathway, and could serve as a liquid biopsy marker to monitor the progress of cancer patients (Liu P. et al., 2019). In CRC, circCCT3 was a potential biomarker of advanced disease (Li et al., 2020). CCT3 was also found to be a serum liver cancer biomarker. Seral-CCT3 was increased in liver cancer patients compared to normal patients and correlated with other serum markers like alpha-fetoprotein (AFP) (Liu Y. et al., 2019). A different approach found that NSCLC patients produced auto-antibodies to CCT5 at higher levels than healthy individuals; so, screening for these auto-autoantibodies in sera could be a novel diagnostic for cancer (Gao et al., 2017). CCT subunit proteins were found in extracellular vesicles or exosomes secreted by cancer cells. Exosomes from neurosurgical aspirates taken from patients with glioblastomas contained all eight CCT subunit proteins (Hallal et al., 2019). The capture and enumeration of circulating tumors cells from blood is another promising liquid biopsy approach, but is only approved by the FDA for breast, prostate, and colon cancer (Miller et al., 2010). Using CCT2 as a marker to identify circulating tumor cells in blood enhanced the ability to detect these cells from a broader range of cancers like small cell lung cancer (SCLC) that currently lack non-invasive diagnostics for patient management (Cox et al., 2022).

## Summary and Open Questions

The evolution of CCT in eukaryotes endowed the chaperonin with remarkable properties of plasticity to handle complex proteins along with the specificity required to produce essential cytoskeletal proteins. New research revealed novel substrates and functions that challenged the concept of CCT as only a protein folding complex for actin and tubulin. The work of many labs, beginning with the first structural studies in the 1990’s, brought to light unique aspects of the chaperonin that are key to the etiology of diseases like cancer. However, gaps in knowledge need to be addressed to fully explore the therapeutic targeting of CCT. How large is the CCT interactome? *In silico* and affinity capture/mass spectrometry studies suggest that there are hundreds of proteins that could interact with CCT subunits. Demonstrating that these lower abundance interactors are directly bound by CCT requires further experimentation. Is ATP hydrolysis required for all the activities mediated by CCT? There are ATP-independent activities, such as those involving the apical domain of CCT1 (e.g., ApiCCT1) (Sontag et al., 2013) or the folding of AML1-ETO (Roh et al., 2016) that suggest otherwise. Different ATP-binding affinities for CCT subunits indicate that the chaperonin may function under a range of ATP concentrations, such as the nutrient-deprived tumor environment; however, findings on the role of CCT4 as an ATP sensor highlight the complex regulation of the ATPase cycle (Jin et al., 2019). Can non-folding activities of CCT be ascribed to subunit monomers or the hetero-oligomer? Nuclear CCT subunits were reported in early studies (Roobol and Carden, 1999), and unique monomer functions such as the interaction of the CCT5 monomer with MRTF-A are known (Elliott et al., 2015). The fact that CCT may interact with some substrates differently from actin or tubulin complicates the question of unique monomer/subunit activities. For example, CCT interacts differently with the actin filament severing and capping protein, gelosin, as compared to actin. Gelosin is not a classical folding substrate of chaperonin (Brackley and Grantham, 2011). In fact, CCT appears to sequester activated gelosin in the complex’s central cavity to protect it from caspase-3 cleavage but does not fold gelosin (Cuellar et al., 2022). CCT may perform functions beyond protein folding that explain its activity in diseases like Huntington’s or Alzheimer’s or cancer. Do subunits like CCT2 or CCT5 have unique activities? The genes for these subunits are often amplified in cancers and correlate with poor patient prognosis. To answer this, new information on the transcriptional regulation of CCT subunits and the processes controlling assembly and disassembly of the complex may be needed. Unlike HSP70 and HSP90 (Backe et al., 2020; Nitika et al., 2020) in which proteomic-based investigations revealed the effects of PTMs on the chaperones’ activities, much remains to be learned about how PTMs, like phosphorylation (Abe et al., 2009), acetylation, palmitoylation (Blanc et al., 2015), or others could modulate the function of CCT subunits. The activities of CCT go beyond neurological disorders and cancer and impact other diseases like diabetes, Down’s Syndrome, hepatitis, and even pain syndrome (Table 1). As an example, targeting the malarial CCT complex with the antihistamine clemastine could be a new therapeutic approach for this parasitic disease (Lu et al., 2020), and CCT in T cells also promotes an immune response against helminths (Oftedal et al., 2021). Even though the gene encoding CCT was first reported in 1979, exploration of the complex activity of this eukaryotic chaperonin is just beginning. The initial work of researchers on the structure and function of CCT provided the foundation for the questions asked today on the role of chaperonins in disease, answers for which lie with current and future investigators working to understand the puzzle that is the CCT chaperonin.
